# Atmospheric Chemistry Insights from the Global COVID-19 Pandemic: A Review

**DOI:** 10.1021/acs.est.5c16737

**Published:** 2026-04-01

**Authors:** Colette L. Heald, Jesse H. Kroll, Jennifer G. Murphy, Delphine K. Farmer, Juliane L. Fry

**Affiliations:** † Institute for Atmospheric and Climate Science, 2167 ETH, Zurich 8092, Switzerland; ‡ Department of Civil and Environment Engineering, MIT, Cambridge, Massachusetts 02139, United States; § Department of Chemistry, University of Toronto, Toronto M5S 3H6, Canada; ∥ Department of Chemistry, 3447 Colorado State University, Fort Collins, Colorado 80523, United States; ⊥ Meteorology and Air Quality Group, 6686 Wageningen University, Wageningen 6700 HB, Netherlands

**Keywords:** atmospheric chemistry, air quality, COVID-19, pandemic, ozone, PM_2.5_, secondary pollutant

## Abstract

The COVID-19 pandemic and resulting reductions in worldwide emissions, associated primarily with the transport sector, provided an unprecedented opportunity to explore the response of atmospheric chemistry and composition to large anthropogenic emissions perturbations. While air quality generally improved in early 2020, this was tempered by increased formation of secondary pollutants (e.g., O_3_ and secondary particulate matter, PM) in many regions studied. Declines in NO_*x*_ emissions were largely responsible for the changes in O_3_, driving decreases in O_3_ concentrations in remote regions and increases in urban regions due to both decreases in O_3_ titration by NO_*x*_ and also nonlinear changes in O_3_ production. Lower NO_*x*_ levels also increased the levels of other oxidants (e.g., OH and O_3_), leading to a general increase in atmospheric oxidation in polluted urban regions. This enhanced oxidation promoted additional PM formation in some regions but was generally outweighed by decreases in primary PM and other secondary precursors (SO_2_ and VOCs). The COVID-19 pandemic gave rise to large local perturbations in air quality but only modest reductions in the global abundance of short-lived climate forcers (including O_3_ and PM).

## Graphical abstract

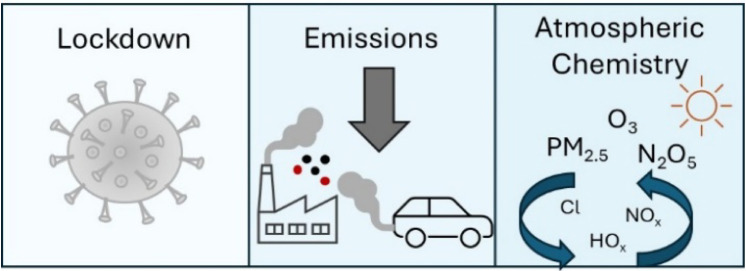

## 1 Introduction

Air pollution degrades visibility, is ruinous to human health, and can alter global climate.^1,2^ The concentration of reactive gases and particles in the atmosphere is controlled by a complex, nonlinear, and highly coupled cascade of processes (emissions, chemical transformations, physical removal). This complexity can obscure the relationship between emissions and atmospheric composition, complicating the crafting of effective environmental policies. In particular, evidence for the health risks associated with outdoor air pollution is strongest for PM_2.5_ and O_3_, which are predominantly secondary (or chemically formed) pollutants.

The COVID-19 pandemic in 2020 and the attendant worldwide reductions in anthropogenic emissions were an unparalleled global perturbation, providing an opportunity to examine how atmospheric chemistry and composition responds to large emissions reductions. Thus, this case study may anticipate the consequences of future air pollution policies (e.g., vehicle electrification) and impart novel insights into the chemical mechanisms at work in a cleaner atmosphere.

Air pollutant changes were widely reported during the COVID-19 pandemic; several reviews have summarized the observed departures in emissions and routinely measured air pollutants.^3–6^ Despite the dramatic drop in emissions during this time, air pollution worsened in some regions. These consequences, while seemingly counterintuitive, were in many cases the result of known nonlinear atmospheric chemistry. In a comment piece published in 2020, we underscored the chemical complexities of air pollution and the research opportunities represented by the pandemic.^7^ We highlighted the following four fundamental components of the atmospheric chemical system that can be informed by studying the COVID-19 response:

1. **Key Emissions:** What is the influence of specific chemical compounds or classes on local O_3_ and PM_2.5_ formation?
2. **Chemical Regime:** How do changes in emissions influence oxidant levels, peroxy radicals, and local chemical regimes? What effects do these have on secondary pollutants?
3. **PM**_**2.5**_ **Chemistry and Impacts:** How have number concentrations, mass concentrations, and chemical composition of particulate matter changed? Do such changes have an impact on the toxicity or cloud-forming potential of the particles?
4. **Global Atmosphere:** Are changes to atmospheric composition limited to urban/polluted regions, or do they extend to remote/pristine ones as well?

In this Review, we explore what studies focusing on the COVID-19 pandemic revealed about air pollution from the local to the global scale. We note that the effects of the pandemic were most strongly felt in early 2020 (boreal spring), and thus the air quality response, as described in the studies reviewed here, reflects the relatively weak photochemical conditions of that season. Comparable emissions changes in a different season may have had a markedly different effect.^8^ We focus on how these studies inform our understanding of atmospheric chemistry and look to answer the above questions we posed 6 years ago. Developing local strategies for cleaner air and meeting the tightening global air quality targets set forth by public health agencies (e.g., the World Health Organization) requires a fuller understanding of the myriad sources of atmospheric pollutants and the processes that control their atmospheric abundance. Emissions changes arising from the COVID-19 pandemic provided an opportunity to test and develop this understanding.

## 2 Overview of Emissions and Observed Surface Air Quality Perturbations

The COVID-19 global pandemic spread first through China and then worldwide in early 2020. Stay-at-home policies during the COVID-19 pandemic curtailed emissions associated with road transport, aviation, and the commercial sector.^9–12^ These were counteracted, to some degree, by increases in residential heating and cooking emissions. Industrial emissions generally declined, but were estimated to increase in China after March 2020.^13^ The degree and timing of the emissions changes associated with the pandemic were strongly dependent on the stringency of local lockdown policies and human mobility (e.g., the Chinese New Year). In some regions, vehicular emissions dropped by up to 70%.^11^ Figure 1 shows the worldwide estimated April 2020 changes in anthropogenic emissions of nitrogen oxides (NO_*x*_) and nonmethane volatile organic compounds (NMVOC), compared to April 2019. We choose April 2020 as the month with the largest global estimated decrease in emissions, but note that in China emissions decreases were larger in February–March. These estimates were made by scaling bottom-up emissions inventories by mobility, industrial, and power generation activity data. The availability and accuracy of these data are regionally variable, contributing to the reasonably high uncertainty on resulting emissions totals. Doumbia et al.^11^ estimate that uncertainties on their emissions scalings range from ±10–30% depending on region and sector. In addition, we note that emissions differences shown in Figure 1 are not constrained by observed concentrations changes. Among reactive gases and particles, declines in emissions of anthropogenic NO_*x*_ were most pronounced, largely in response to decreased vehicular use, with global emissions estimated to have dropped by 18–31% in April 2020. These changes were not always uniform: for example earlier lockdowns in China produced an overall decrease in anthropogenic NO_*x*_ emissions in February–March, while emissions increases in the industrial sector offset the declines in transport and commercial emissions in April 2020. Global anthropogenic NMVOC and carbon monoxide (CO) emissions fell similarly by 19–27%, though decreases in NMVOC emissions (Figure 1a) were estimated to be more modest than those of NO_*x*_. Global emissions reductions of other species, including sulfur dioxide (SO_2_) and black carbon (BC), were more modest (10–23%). Estimates differ as to whether primary organic carbon (OC) emissions decreased^10,12^ or increased,^11^ the latter due to dominant (and elevated) residential contribution to CO emissions.

**Figure 1:**
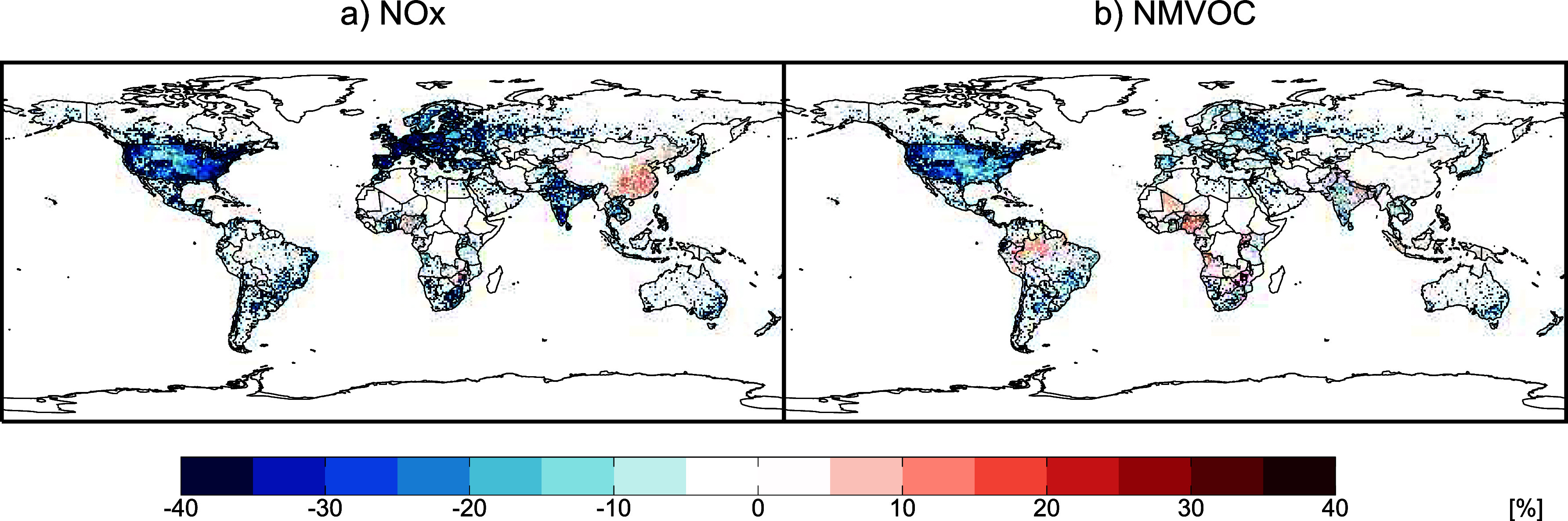
Percentage changes in April 2020 anthropogenic emissions of NO*_x_* (a) and NMVOCs (b), during the COVID-19 pandemic, compared to April 2019. Emissions for 2019 are taken from the CAMS v6.2 data set.^13^ Sectoral adjustments factors estimated by Doumbia et al.^11^ are applied to estimate 2020 emissions. Color bar is saturated at respective values.

The resulting observed deviations in air quality experienced during the spring of 2020 need to be carefully analyzed in the context of seasonal and long-term pollution trends, as well as the impact of year-to-year variations in meteorology, and generally cannot be wholly attributed to emissions changes (e.g. ref 14). Published COVID-19 air pollution studies are inconsistent in terms of estimating or accounting for meteorological variation. Wang et al.^15^ provide a comprehensive discussion of the various detection and attribution methods applied to estimate the fingerprint of COVID-19. We do not review the meteorological impacts on air quality in 2020 in detail here, but in what follows make note of studies that ascribe a dominant role for meteorology, particularly as it impacts atmospheric chemistry. The majority of the studies reviewed focus exclusively on springtime differences (i.e., boreal spring 2020 compared to previous springs); studies that focus on other time scales are noted.

The dramatic changes in anthropogenic emissions in the spring of 2020 produced corresponding changes in the concentrations of primary pollutants. (However, as discussed below, atmospheric chemical reactions can complicate the relationship between emissions and concentrations.) Numerous studies have documented or reviewed the observed concentration changes of key species.^3,5,6^ These studies generally focus on urban to suburban locales, where the preponderance of monitoring stations are located, and where vehicle emissions reductions were highly influential. In this section we summarize those observed changes, based primarily on the comprehensive global-scale review of Gkatzelis et al.,^3^ which summarizes both *in situ* and satellite measurements averaged to the national and regional scale. Quantitative values in this section come from this study unless stated otherwise.

In spring of 2020, observed urban concentrations of primary (i.e., directly emitted) species declined precipitously. Reductions in NO_2_, a species that is clearly seen from space, were particularly well documented. TROPOMI-derived population-weighted global surface NO_2_ concentrations fell by 16% in 2020 relative to 2019.^16^ Median NO_2_ measured at ground-based measurement stations dropped by 20–70% across the world compared to previous years, with similar estimated NO_*x*_ declines. At the national scale, the percentage decline in NO_2_ concentrations generally agrees with the emissions cuts estimated by Forster et al.,^10^ within a factor of 2. However, the local relationship between NO_*x*_ emissions and NO_2_ concentrations depends critically on the NO_*x*_ lifetime and the [NO_2_]/[NO] ratio (see discussion in Section 3).^17^ In the most well-studied countries, median NO_2_ surface concentrations during the COVID-19 perturbation were lower by 40% (Italy), 23% (USA), 32% (China), and 42% (India). SO_2_ and CO median concentration decreases ranged from 5 to 49% and 16–49%, respectively. A limited number of studies reported median declines of 51% and 19% for BC and NH_3_. All of these pollutants exhibited median regional decreases, but some individual studies reported higher concentrations during the COVID-19 pandemic, most notably in South Asia.

It is more challenging to develop a comprehensive view of how COVID-19-related reductions in anthropogenic activities impacted atmospheric NMVOC concentrations, largely because routine measurements of these species are rare, and often only include a small number of species or some subset of NMVOCs, such as nonmethane hydrocarbons (NMHCs). Furthermore, the NMVOC moniker includes a range of both primary and secondary species, which may be controlled by different drivers and sources (both anthropogenic and biogenic). However, it is clear that decreases in NMVOC concentrations during COVID-19 were generally smaller than those of NO_*x*_. Total NMHC or VOC concentrations were reported to decrease by 14–25% in Rio, Brazil^18^ (compared to prelockdown), 20–60% in Leicester, UK^19^ (compared to prelockdown), and 32% in Chengdu, China^20^ (compared to same time interval in the previous 2 years). Jensen et al.^21^ estimate that VOC concentrations associated with all sectors (industry, traffic, secondary chemistry) decreased by 20–71% during lockdown in Changzhou, China (compared to prelockdown). Concentrations of aromatics, including benzene, toluene, and xylenes, which are primary and largely anthropogenic, declined at surface sites in South India, Milan, the UK, and in the Yangtze River Delta (YRD) region of China.^19,21–24^ Jia et al.^23^ reported that while aromatics declined in YRD, concentrations of alkenes increased. Tan et al.^20^ similarly reported an increase in alkenes, despite an overall decrease in AVOCs (anthropogenic VOCs) during lockdown in Chengdu, China. Polycyclic aromatic hydrocarbon (PAH) concentrations also dropped by up to a half at a background monitoring site in Japan.^25^ Satellite observations of formaldehyde, a VOC with both primary and secondary sources that is sometimes used as a proxy for VOC emissions or oxidation, revealed modest decreases (8–22%) in Asia during the pandemic.^26^ Formaldehyde can be produced from biogenic as well as anthropogenic sources, thereby dampening the perturbation associated with the COVID-19 pandemic. Parker et al.^27^ suggested that the observed 10% decrease in formaldehyde concentrations over Pasadena, California (compared to the previous 5 years) was consistent with a 30% reduction in VOC emissions during the COVID-19 period.

Ozone is the only pollutant that increased at the majority of urban surface sites during the COVID-19 pandemic. Around the world, the observed median O_3_ response ranged from a 15% decrease to a 20% increase. As a secondary pollutant, the effect of precursor emissions on O_3_ depends critically on the chemical environment. We review how changing chemical regimes and oxidants impacted O_3_ concentrations during COVID-19 in Section 4.

During the COVID-19 pandemic, visibility markedly improved in some regions of the world, particularly in South Asia, largely due to decreases in atmospheric aerosols. Globally, median observed PM_2.5_ mass concentrations dropped by 10–40%; with modest reductions in Europe and greater reductions in Asia. Exceptionally, reported PM_2.5_ concentrations increased in Paris and London.^14^ PM_2.5_ comprises both primary and secondary particles, produced from a range of sources, complicating the response to the pandemic-induced emissions changes. We review what has been learned about how COVID-19 perturbed the chemical formation and composition of PM_2.5_ in Section 5.

## 3 How Atmospheric Chemistry Responds to Emissions Perturbations

This section provides an overview of the tropospheric chemistry principles relevant to interpreting the studies reviewed in Sections 4–6. We also present a conceptual framework describing how anthropogenic emissions decreases can impact O_3_ and PM.

Figure 2 illustrates the basic radical cycling in the troposphere, showing the central role of NO_*x*_ and how it links to the key atmospheric oxidants (OH, O_3_, NO_3_, Cl). NO can be oxidized to NO_2_ by O_3_, with subsequent photolysis of the NO_2_ producing an O atom that regenerates O_3_. While these reactions are effectively a null cycle, Leighton^28^ showed that under steady state conditions, the concentration of O_3_ scales with the ratio of [NO_2_]/[NO]. A corollary is that emissions of NO will react with O_3_ to produce an equivalent amount of NO_2_, motivating the concept of odd oxygen ([O_*x*_] = [O_3_] + [NO_2_]), a quantity that is invariant even when fresh NO perturbs the null cycle (left side of Figure 2). In parallel, when NO is oxidized by HO_2_ or RO_2_ radicals, followed by NO_2_ photolysis, this catalytic cycle results in net O_3_ production and an equivalent increase in [O_*x*_] (dashed box in Figure 2). Catalytic ozone production (P_O3_) depends nonlinearly on the concentrations of NO_*x*_ and VOC precursors, which can be diagnosed by the fate of the HO_*x*_ radicals. Under NO_*x*_-limited conditions, the dominant fate of HO_2_ radicals is self-reaction to form H_2_O_2_, and increases in NO_*x*_ promote the catalytic cycling to generate O_3_. Under NO_*x*_-saturated, or VOC-limited, conditions, high concentrations of NO_2_ react with OH to form HNO_3_; further additions of NO_*x*_ increase this termination, reducing O_3_ formation. Under these conditions, concentrations of OH are suppressed, slowing the oxidation of VOCs. The NO_3_ radical (a strong oxidant) is produced by the reaction of NO_2_ with O_3_, but during the day is lost quickly to photolysis. At night, NO_3_ can react with VOC to form organonitrates or HNO_3_, or react with NO_2_ to form N_2_O_5_; however, even small amounts of NO will recycle the NO_3_ back to NO_2_. When N_2_O_5_ is taken up on chloride-containing aerosol, the production of volatile ClNO_2_ can lead to a source of Cl radicals (themselves strong oxidants) after sunrise.

**Figure 2:**
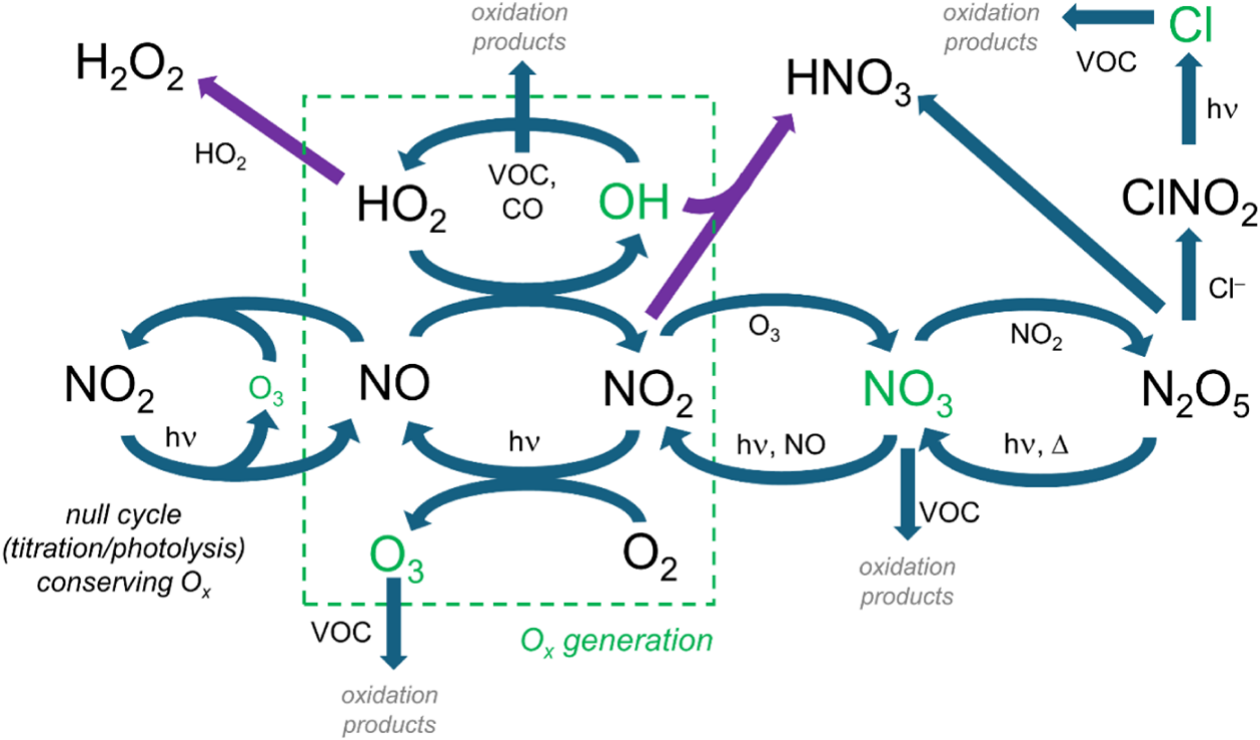
Illustration of radical cycling in the troposphere, emphasizing the role of oxidants (in green). Shown in purple are the termination of the HO*_x_*–NO*_x_* cycling via formation of HNO_3_ or H_2_O_2_. Organic peroxy radicals (RO_2_), formed from VOC oxidation and lost by a number of reactions (including reaction with NO to form O_3_), are omitted for clarity.

Figure 3 summarizes how anthropogenic emissions perturbations, such as those experienced during the COVID-19 pandemic, alter the key pollutants O_3_ and PM; this figure serves as a roadmap for the secondary chemistry to be discussed in Sections 4, 5, and 6. Figure 3 presents two limiting (and idealized) cases: changes to “unpolluted” areas (i.e., rural or remote regions, far from pollution sources and characterized by low baseline NO_*x*_ levels), and changes to “polluted” areas (i.e., urban regions, which have high local sources and hence are characterized by high baseline NO_*x*_). We note that there exists a myriad of intermediate environments, and indeed in some cases, emissions reductions during the COVID-19 pandemic may have moved a regime from “polluted” to “less polluted”.

**Figure 3:**
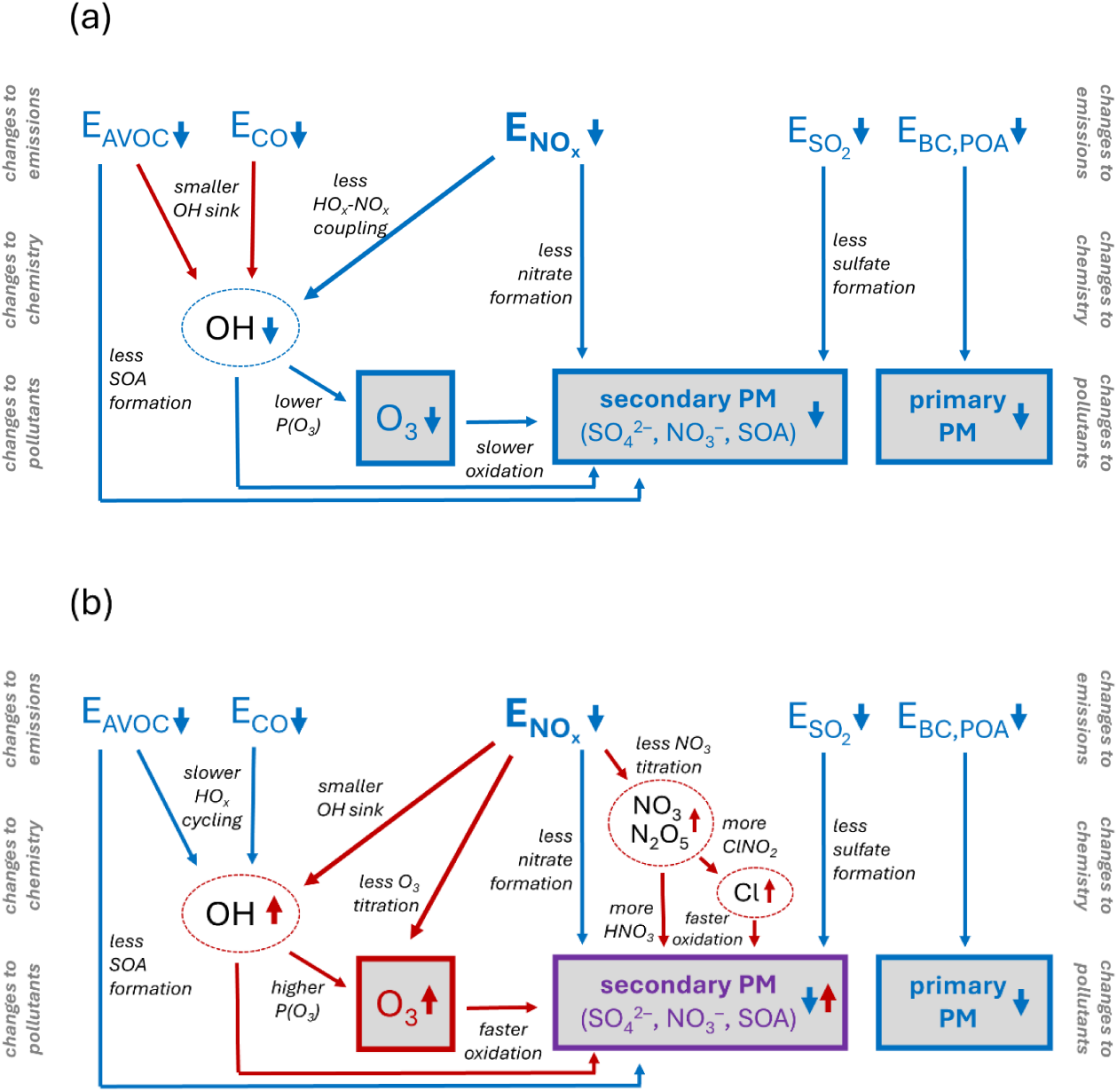
Schematic describing how anthropogenic emissions changes may perturb O_3_ and PM concentrations in (a) unpolluted and (b) polluted conditions. Color indicates the direction of the changes: blue denotes a decrease, red an increase, and purple a possible mixed response. These two regimes represent limiting conditions; many environments will be in between the two (“transition regimes”), and thus the operative chemical effects may be a mix of the ones shown here. All effects shown are based on the chemistry described in Figure 2, and excludes other potential chemical effects or feedbacks, such as changes to UV fluxes and hence oxidant levels; changes to HO_2_ loss by uptake to PM; altered HO*_x_* production by CH_2_O and other precursors; changes to RO_2_ chemistry; shifts in aerosol partitioning (e.g., changing ammonia partitioning due to lower sulfate and nitrate); changing aerosol water content and aerosol pH and resulting changes in multiphase chemistry; and NO*_x_*-dependent changes to SOA yields.

As discussed above, among all species and across all regions, NO_*x*_ emissions were most consistently and substantially altered by the COVID-19 pandemic. The resulting NO_*x*_ concentration changes shaped the chemical responses that are largely responsible for the observed changes in secondary pollution in spring of 2020. Figure 3 illustrates how changes in NO_*x*_ drive much of the complex response in secondary pollutants, and that this response is dependent on the underlying chemical regime. While a reduction in NO_*x*_ largely translates to less ozone and secondary PM under “unpolluted” conditions, under “polluted” conditions NO_*x*_ reductions drive increases in ozone and secondary PM. These NO_*x*_-driven increases in PM can be counterbalanced by coincident sectoral decreases in anthropogenic SO_2_ or VOC emissions. For decades, atmospheric chemists have sought to use variability in NO_*x*_ emissions arising from changes in activity patterns to diagnose the local NO_*x*_-dependence of ozone production. In North America and Europe, significant reductions in NO_*x*_ emissions on the weekend have historically provided an opportunity to assess ozone production regimes (e.g., refs 29, 30 ), whereas in China, NO_*x*_ emissions reductions on weekends can be more muted.^31^ Prior to the COVID-19 pandemic, the 2008 Olympics in Beijing provided one of the only other opportunities to assess changes in air quality caused by a large reduction of emissions in China.^32^

## 4 Chemical Regimes and Oxidants

The abrupt decreases in NO_*x*_ emissions during the COVID pandemic (and in the emissions of other anthropogenic species) had the potential to shift different regions into new atmospheric chemical regimes (i.e., from “polluted” conditions to “unpolluted” conditions, Figure 3). Such shifts might lead to changes in oxidant levels and concentrations of secondary pollutants (O_3_ and PM); these changes provide insight into the dependence of key atmospheric transformations (Figure 2) to emissions, as well as to the chemical regime a given area was in prior to the lockdowns.

The COVID-era O_3_ response varied from location to location (Section 2). In “unpolluted regions” (regions well outside of urban areas, including most of the global/remote atmosphere), O_3_ decreased.^33–35^ This decrease was expected since most of the remote atmosphere is NO_*x*_-limited, so lower NO_*x*_ is expected to lead to lower OH levels and slower O_3_ production (Figure 3a); this oxidant effect was captured by regional and global modeling studies.^36,37^

Within urban/polluted areas, where most COVID-era analyses of changes to air quality were made, ozone levels often increased.^3^ As shown in Figures 2–3, this can arise from two primary factors, the increase in P_O3_ as a result of higher OH levels (which in turn result from lower NO_2_) and the decrease in O_3_ titration arising from lower NO. This titration can dominate on the local urban scale, as high NO levels will lower O_3_ concentrations, leading to decreased exposures of city residents to O_3_; however, this sequestration of O_3_ as NO_2_ is only temporary, and so downwind (on the regional scale) the increase in P_O3_ is likely to be the dominant effect.

Studies of changes to O_3_ during the COVID pandemic distinguished these two chemical effects via the analysis of odd oxygen (O_*x*_), which removes the effect of titration. In general, changes in O_*x*_ were substantially smaller than changes in O_3_;^20,27,38–40^ in some cases, changes in O_*x*_ and O_3_ were even of the opposite direction, with ozone increasing (due to less titration) despite a decrease in P_O3_ leading to lower O_*x*_ (once decreases in NO_*x*_ shifted the ozone production regime to be NO_*x*_-limited).^41^ These results underscore the importance of titration in governing the response of urban ozone levels to NO_*x*_ emissions changes.^41–43^ Had the largest perturbations to NO_*x*_ emissions occurred during a warmer or more photochemically active period, the relative importance of changes in ozone production may have been much stronger at several study locations.

Nonetheless, studies that take titration into account did find that in some locations, reduced emissions during the COVID-19 pandemic indeed led to changes to P_O3_ and shifts in the overall ozone-formation regime. H. Parker^27^ and L. Parker^38^ used observation-based and modeling analyses, respectively, of pre- and lockdown conditions to identify different portions of the South Coast Air Basin (SoCAB) of California as residing within NO_*x*_-limited and VOC-limited production regimes. Using satellite-derived HCHO/NO_2_ ratios, and analysis of the weekday–weekend effect in air quality monitoring data, Schroeder et al.^44^ argued that P_O3_ evaluated across the SoCAB as a whole became NO_*x*_-limited in 2020 and emphasized the importance of pursuing further NO_*x*_ emissions reductions. Similar effects were observed over northeastern China: satellite-derived HCHO/NO_2_ ratios and model analysis suggest that during the pandemic period, a substantial fraction of the region that had been in the VOC-limited production regime shifted to transitional conditions, and even became NO_*x*_-limited.^36,41^ However, not all cities exhibited such shifts in ozone formation regime: ozone production increased during the pandemic within cities in India,^40^ China,^20^ and Europe,^39^ suggesting that many intensely polluted areas remained in the VOC-limited regime despite the decreases in NO_*x*_.

Complicating these analyses are additional effects that may also have led to changes in O_3_ levels, some (but not all) of which are controlled by changes in NO_*x*_: these include increases in ozone production efficiency (the number of O_3_ molecules formed per NO_*x*_ molecule), which increases as NO_*x*_ decreases;^34,45^ and changes to the rate of O_3_ destruction in the upper troposphere.^46^ However, the roles that these various proposed effects had on O_3_ have not been compared systematically, so it is difficult to assess the relative importance of each.

Substantial work has focused on changes to levels of atmospheric oxidants, particularly OH, during the COVID-19 pandemic: this is important not only for ozone formation but also for the formation of other secondary species, and for lifetimes of other key pollutants of interest. In the free troposphere and globally, 3D modeling suggests OH levels were lower in spring 2020 (2–5% globally averaged).^35,47–49^ Changes to OH concentrations are driven not only by decreases in NO_*x*_, but also by decreases in CO and NMVOC, which can serve as important OH sinks (Figure 2).^35,47^ Reifenberg et al.^47^ note that while OH decreased by ∼5% in the free troposphere over Europe in their model, a greater ∼20% drop was simulated in the upper troposphere corresponding to reduced aircraft emissions. A lower global [OH] leads to a longer methane lifetime and hence may partially explain the recent surge in methane concentrations that began in 2020;^4^ however greater methane emissions appear to have contributed to this increase as well.^50–55^ Model simulations suggest that the COVID-19-driven decreases in OH would increase methane lifetime by only 2–3% over a short interval, with little impact on methane concentrations.^35,48^ The exact balance between the decreased OH sink and increased sources on methane remain unclear.

In urban environments, many studies (mostly carried out in cities in China) suggested that lockdowns led to higher OH levels and oxidation rates,^20,36,41,56,57^ both of which are sometimes referred to as “atmospheric oxidation capacity” (AOC). Most such changes were a result of the lower NO_2_ levels, which led to a smaller OH sink. However, in some cases, increased sources of HO_*x*_ (via increased HCHO photolysis^58^ or alkene ozonolysis^20^) may also have contributed to higher OH. (These are oxidant amplification mechanisms: more OH or O_3_ led to increased VOC oxidation, which in turn led to higher HO_*x*_ production, increasing oxidant levels still further.^20^) In addition, the lower PM levels may have decreased loss of HO_2_ via uptake to PM^58^ and also increased the rates of photochemical reactions due to higher UV fluxes.^40^ The resulting increases in OH can explain not only higher O_3_ production rates, but also the greater production of secondary PM_2.5_ (SOA, sulfate, nitrate) observed in some cities^41,56,58^ (see Section 5). At the same time, in some cases the lower emissions of primary species that are subject to oxidation (e.g., NMVOCs, SO_2_) may have somewhat counteracted the increase in OH, leading to modest changes in total oxidation.^27^ To our knowledge, there were no direct measurements of changes to OH concentrations within urban areas. However, using satellite NO_2_ measurements downwind of six cities, Lama et al.^59^ found that during the pandemic periods, NO_2_ lifetimes decreased, consistent with increases in OH.

Increases in oxidants other than OH and O_3_ during the COVID-19 pandemic were also inferred. Yan et al.^57^ used observations of elevated levels of N_2_O_5_ and ClNO_2_ in Beijing during the pandemic to infer dramatic increases in nighttime NO_3_ and daytime Cl radical concentrations (right side of Figure 2). They attributed these changes to substantially decreased NO emissions, which prevented the titration both of O_3_, a key precursor to NO_3_, and of NO_3_ itself, an impact also noted by others.^56,60^ As with increased OH, increased NO_3_ and Cl can then promote the formation of secondary aerosol (most importantly inorganic nitrate and SOA).^57^

Fewer studies have examined how the pandemic-related decrease in anthropogenic emissions may have perturbed the chemistry of organic peroxy radicals (RO_2_), key intermediates and branch points in VOC oxidation processes.^61^ A decrease in NO_*x*_ may affect RO_2_ chemistry in that it will shift reactions away from RO_2_ + NO reactions and toward other pathways (RO_2_ + HO_2_, RO_2_ + RO_2_, and RO_2_ isomerization), which in turn can affect radical cycling and VOC product distributions. Indeed, Nussbaumer et al.^46^ showed that during the pandemic, the lower NO_*x*_ levels in the upper troposphere had a marked influence on the branching of the methylperoxy radical (CH_3_O_2_, the simplest and most abundant RO_2_), with a smaller fraction reacting via RO_2_ + NO, leading to generally slower radical cycling. To date, only one study has explicitly examined RO_2_ chemistry within an urban environment: Yan et al.^62^ found that the composition of oxygenated organic molecules (OOMs) in Beijing were largely unaffected by the COVID-19 pandemic, indicating little shift in RO_2_ chemistry (or other branch points in VOC oxidation mechanisms). This is presumably because of the very high levels of NO_*x*_ to begin with; it is possible that in locations with lower baseline NO_*x*_ levels, RO_2_ chemistry did undergo substantial shifts, but we are unaware of any studies examining RO_2_ chemistry in such environments.

## 5 Changes to Surface PM_2.5_ Chemistry and Impacts

Changes to emissions and chemical regimes during the COVID-19 pandemic also led to substantial changes to fine PM_2.5_, a major component of anthropogenic air pollution. Below, we summarize research on the response of surface PM_2.5_ mass, composition, number and toxicity to COVID-19 induced emissions changes. We note substantial literature on linkages between ambient PM_2.5_ exposure and COVID-19 incidence, severity and mortality, but consider that topic beyond the scope of this review.^63^

Coupled analyses of satellite data and modeling demonstrated the response of surface fine particulate matter (PM_2.5_) mass loadings to reductions in anthropogenic emissions around the globe, but with distinct regional characteristics. For example, combining satellite-derived aerosol optical depth (AOD) measurements with ground-based observations and a global model, Hammer et al.^64^ found that surface PM_2.5_ in different regions had different sensitivities to primary emissions. China experienced the largest population-weighted mean PM_2.5_ concentration reductions of −11 to −15 μg m^–3^, though changes to PM_2.5_ were far smaller in Europe (+1 to −2 μg m^–3^) and North America (0 to −2 μg m^–3^). We interpret this regionality as likely driven by different relative reductions in transport emissions, resulting in different NO_*x*_ decreases and consequent secondary chemistry. NO_*x*_ is a driver of nitrate aerosol formation, but, depending on the regional oxidation regime, decreases in PM_2.5_ nitrate could be offset by increases in sulfate or secondary organic aerosol (Figure 3).

The chemical nonlinearities inherent to atmospheric chemistry complicate direct linkages between emissions reductions and aerosol mass loading. However, examination of individual aerosol species can provide more insight into the chemical regime of a given region. Gaubert et al.^37^ found that despite calculated increases in hydroxyl radicals and thus more atmospheric oxidation, surface-level secondary organic aerosol (SOA) concentrations decreased by 10–30% in China, the Americas, Europe and India in their global simulation in response to reductions in NO_*x*_ and VOC emissions (Figure 3b). Notably, this model suggested that if only NO_*x*_ emissions had been reduced, the subsequent increase in OH radicals would have caused SOA to increase in some areas by up to 20%. Using a different model framework, Sekiya et al.^65^ suggested a global reduction of 8–21% in sulfate and nitrate aerosols over polluted regions during April 2020 due to reduced SO_2_ and NO_*x*_ emissions, and an 11–17% reduction in ammonium aerosol despite unchanged NH_3_ emissions, highlighting the interconnected nature of acidic and basic aerosol components. A satellite-based inverse modeling study in Europe found a 10% decrease in ammonia emissions, despite a slight increase in observed ammonia surface concentration.^66^ This can be explained by coincident reductions in SO_2_ and NO_*x*_ emissions, which reduce NH_3_ losses and thus increase its lifetime. Furthermore, it was found that the high availability of gaseous NH_3_ in Europe “buffers” PM_2.5_ levels, due to the equilibrium shift toward particulate nitrate, which slows deposition. This explains the observations of unchanged or even increased PM_2.5_ levels in some locations in Europe, despite decreases in precursor inorganic emissions. Widespread, continuous observations of aerosol composition would be useful for validating these model findings, but a lack of long-term monitoring and the challenge in treating existing data consistently may be hindering such an analysis in many regions of the world.

The complexity and nonlinearities driven by NO_*x*_ reductions were made particularly apparent in a series of independent papers from China. For example, increased oxidant concentrations from decreased anthropogenic emissions strongly enhanced secondary inorganic aerosol and drove air pollution events (Figure 3b). Studies provide evidence for increases in both OH-induced particulate nitrate^67^ and NO_3_- and O_3_-driven particulate sulfate and nitrate^56^ during winter lockdowns in Beijing, with minor effects on secondary organic aerosol. Together, these studies indicate room for continued improvement in wintertime air quality from decreasing emissions of inorganic precursors (i.e., NO_*x*_, SO_2_). In slight contrast, Chang et al.^68^ used a model to further probe secondary organic aerosol formation during winter lockdowns and showed that SOA formation was simultaneously suppressed by decreased emissions of gas-phase organic precursors and enhanced by meteorological effects—resulting in a net increase in SOA in Beijing and a decrease in southern Hebei. Anomalously high humidity during the winter lockdowns in Beijing increased multiphase particulate nitrate formation from N_2_O_5_ hydrolysis, particulate sulfate from aqueous SO_2_ oxidation, and particulate organic from aqueous glyoxal and methylglyoxal formation; Le et al.^69^ modeled this multiphase chemistry and found that it increased PM_2.5_ by 12%. In particular, decreased NO concentrations accelerated haze formation through N_2_O_5_ formation (via NO_2_ + NO_3_ → N_2_O_5_, facilitated by the decrease in the NO_3_ + NO loss channel, Figure 3b).^57^ The subsequent heterogeneous uptake onto particles not only increased particulate nitrate levels but also enabled particle gaseous chlorine chemistry and subsequent SOA formation (Figure 2) the following day.

The changes in PM composition during the COVID-19 pandemic provided the opportunity to explore changes in PM toxicity. Some PM components are more effective at forming reactive oxygen species in biological systems, which then causes oxidative stress and, potentially, health effects. Few studies were able to measure aerosol oxidative potential before, during and, after the COVID-19 pandemic. However, studies in Europe do show that decreased traffic decreased aerosol oxidative potential, but with less or no change in areas with PM dominated by domestic wood burning.^70–72^

Particle size distributions in various urban environments may have been more susceptible than PM_2.5_ mass concentrations to pandemic-related emissions decreases. Putaud et al.^73^ compared urban observations across Europe to ensemble forecasts of what would have been expected without the pandemic and noted that despite negligible change in urban PM_2.5_ mass concentrations, modest decreases in small (<70 nm) particles were apparent, potentially due to decreased private vehicle usage. A decrease in ultrafine (<100 nm) particles was also noted in Beijing,^74,75^ Tianjin,^76^ and Hyderabad.^77^ However, the impact of the pandemic on new particle formation and <3 nm particles remains poorly understood and based on a limited number of studies from two locations. In Hyderabad, no changes in particles <3 nm were noted, suggesting that the formation mechanism was unaffected by reductions in anthropogenic emissions.^77^ In Beijing, one study suggested increased formation rates of 2 nm particles,^75^ while Tang et al.^74^ found no change in formation or growth rates of new particle formation events and Yan et al.^62^ found no change in formation rate of 1.5 nm particles, but increases in growth rates. Thus, effects of dramatic changes in anthropogenic emissions on new particle formation events are subtle and dependent on precise measurement and analysis approaches. As Yan et al.^62^ noted, this increased growth rate of 1.5 nm particles was driven by sulfate and oxygenated organic molecules, but insensitive to NO_*x*_. All three studies from Beijing do agree that new particle formation affects the formation of larger particles and thus plays an important role in controlling severe air pollution events in the region.

## 6 Perturbations to the Global Atmosphere

As described in Section 2, the 2020 COVID-19 pandemic systematically curtailed transport and industrial activity, producing a notable decline in anthropogenic emissions and primary pollutants, particularly in highly populated regions. However, regions and species with large natural sources or anthropogenic sectoral emissions that were unperturbed (or even enhanced) by the pandemic, experienced little (or even opposite) changes. For example, while TROPOMI satellite observations revealed consistent declines in NO_2_ in highly populated regions in spring 2020 compared to previous years, increases were observed in many regions (e.g., central United States, Myanmar, central China, southern Russia, Venezuela, the DRC, northern South Africa).^16^ Furthermore, much of the global atmosphere represents the “unpolluted” conditions of Figure 3a. The impact of the pandemic on the global burden of atmospheric constituents was therefore more muted, and sometimes opposite to, the extreme responses seen at the urban scale.

In 2020, free tropospheric O_3_ measured by sondes in the northern extratropics was on average 7% below the spring and summer mean levels; modeling suggested that the majority of this decrease can be attributed to the COVID-19 emissions perturbation.^33^ Bouarar et al.^78^ also found that the decline was largely associated with anthropogenic emissions changes, but that the Arctic spring-time depletion of stratospheric ozone also played an important role earlier in the season. In 2020, modest declines in free tropospheric ozone were reported from aircraft above Frankfurt^79^ and at the Mt. Cimone free tropospheric site in Italy.^80^ These studies are consistent with lower NO_*x*_ driving lower O_3_ production in the “unpolluted” NO_*x*_-limited remote atmosphere (Figure 3a). Global modeling studies, based on either estimated emissions changes or data assimilation of satellite observations, similarly estimated that the COVID-19 pandemic induced modest global declines of 2–3% in the tropospheric O_3_ burden.^34,48^

Section 5 describes the local and global changes in surface PM_2.5_ during the pandemic. The response of aerosol concentrations in the free troposphere and the global particulate burden were generally more modest, again, largely reflective of the “unpolluted” conditions of Figure 3a. Satellite observations and modeling revealed that most regions, with the exception of India, did not exhibit a statistically significant change in aerosol optical depth (which reflects total column aerosol loading) during the spring of 2020.^81^ Nair et al.^82^ confirmed that satellite measured extinction was 10–40% lower in the free troposphere over South Asia in the Spring of 2020. Sekiya et al.^65^ suggested that VIIRS observed AOD was substantially lower in 2020 over Eastern China and the Eastern U.S., though only the latter exceeded interannual variability. BC and sulfate aerosol measured aloft (below 6 km) during the 2020 BLUESKY campaign over Europe were ∼1/2 of the 2017 EMERGE-EU measurements in the same region, however, nitrate and ammonium concentrations were comparable.^83,84^ Similarly, simulations over Europe showed large relative decreases in BC and sulfate aloft.^47^ Sekiya et al.^65^ showed that sizable reductions in NO_*x*_ and SO_2_ emissions during COVID-19 produced 8–21% decreases in simulated sulfate, nitrate, and ammonium column concentrations over populated regions of the Northern Hemisphere. They showed that these reductions result not only from the direct precursor emission change, but also the indirect effect via oxidants and thermodynamic partitioning (Figure 3, Section 5). At the global scale, Weber et al.^48^ simulated a 2–7% decrease in sulfate burden during the COVID-19 pandemic. Local simulated BC column decreases did not exceed 40%, and globally average to <10%.^48^ Hickman et al.^8^ estimated that the COVID-19 pandemic resulted in a 3.1% decline in global mean AOD in 2020, attributed to a 6.6% reduction in sulfate, 5.9% reduction in ammonium, and 3.6% reduction in carbonaceous aerosol. Several other global models simulated decreases in sulfate and BC during the pandemic but did not provide quantitative estimates of the global mean burden change.^10,65,85^

Given that the global burdens of short-lived climate-relevant species (O_3_ and PM) were only modestly perturbed by the fall of anthropogenic emissions during the COVID-19 pandemic, the associated net radiative forcing and resulting climate response were found to be modest to negligible, and often insignificant.^8,10,48,86^ The COVID-19-induced cooling from ozone was assessed at −0.03 to −0.04 W m^–2^.^10,48^ Gettelman et al.^85^ suggested that global mean aerosol forcing, which they estimate is +0.29 W m^–2^, was the largest forcing from COVID-19 (greater than changes in CO_2_, O_3_, and contrails), though it induced a negligible temperature response. Sekiya et al.^65^ assessed that changes in inorganic aerosol (sulfate, nitrate, and ammonium) corresponded to a global forcing of +0.14 W m^–2^. Forster et al.^10^ found that the decrease in nitrate associated with NO_*x*_ emissions declines induced an insignificant warming. Some studies suggested that the indirect effect of aerosols was negligible compared to the direct radiative effect,^48,65^ while others suggested that the indirect radiative effect was dominant.^47,85^ The largest local climate warming (+1.4 W m^–2^, clear-sky) associated with aerosol reductions were estimated over South Asia.^82^ Globally, studies indicate that the COVID-19 pandemic did not produce a substantial or sustained change in short-lived climate forcers or the climate response to these.

## 7 Summary and Future Perspectives

In the spring of 2020, global anthropogenic emissions declined in response to curtailed anthropogenic activities during the COVID-19 pandemic. The signature of these declines was seen in changes in air quality and atmospheric composition around the world. The simultaneous decrease in emissions across various chemical environments provided a unique case study for exploring secondary pollution formation and the nonlinearities therein, as summarized in this review. Figure 4 summarizes the results from the research to date on this topic as responses to the four fundamental components of the chemical system that we suggested in Kroll et al. (2020).^7^ In particular, the decrease of anthropogenic NO_*x*_ emissions from the transport sector drove myriad complex and interconnected chemical responses. The interplay and relative importance of these processes varied with chemical regime, providing a real-world example of the complexity of atmospheric chemistry. This review provides support to our assertion in Kroll et al. (2020) that it is erroneous to assume that concentrations of atmospheric pollutants respond linearly, or consistently, to changes in emissions.

**Figure 4:**
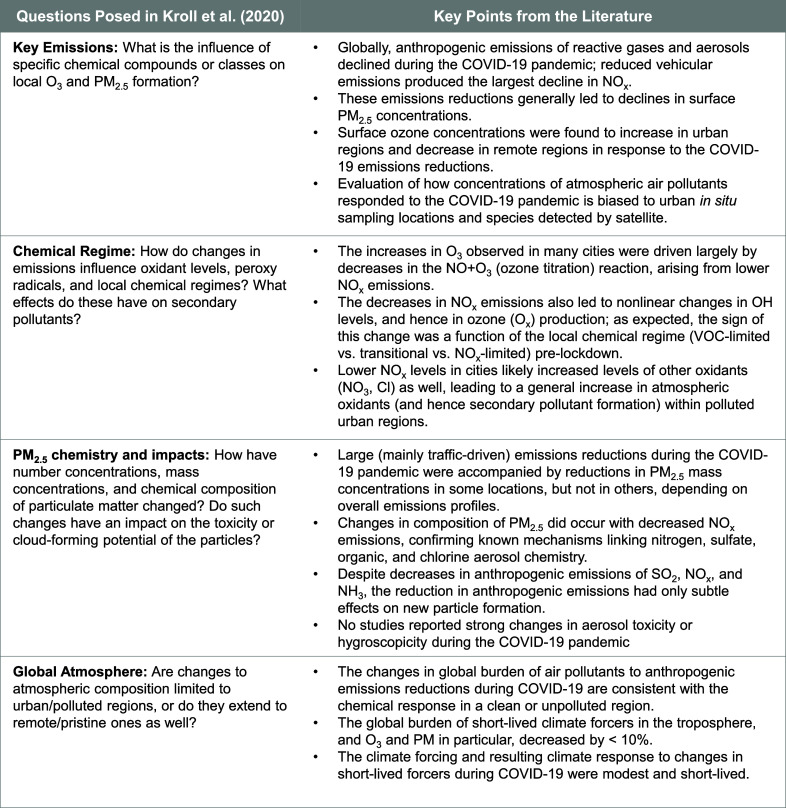
Four fundamental components of the atmospheric chemical system that can be informed by studying the COVID-19 response as raised in Kroll et al. (2020) and the summary of the literature that has explored these questions, and is synthesized in this Review. Please see Sections 2, 4, 5, and 6 for relevant citations.

The COVID-19 pandemic could provide some insights into air pollution management strategies at the local level. For example, the response may be used to anticipate the impacts of future reductions in anthropogenic NO_*x*_ emissions. However, the context for this event is inextricably linked to the response: had the COVID-19 emissions reductions occurred in a different season or indeed year, the response would likely have been markedly different.^8^ Furthermore, in many cases, changes in emissions of other species (SO_2_, VOCs, primary PM) also played a role. Thus, as discussed extensively in this review, the local chemical environment is central to air pollution outcomes, and this includes not only the spatial but also the temporal setting.

The atmospheric chemistry insights afforded by the COVID-19 pandemic relied critically on the availability of measurements both during and preceding (ideally in year(s) prior to) the lockdowns. Thus, many opportunistic field measurements made during the pandemic, but without precedent, were insufficient to characterize the strength of the response. Unfortunately, comparable measurements were not typically continued in the year(s) that followed the height of the pandemic. Furthermore, process-based understanding of how ozone and PM respond to changing emissions requires a suite of research-grade instrumentation to measure radicals, reactive organics, and particle composition that goes well beyond typical air quality monitoring. Thus, studies that were able to characterize the detailed changes in the atmospheric chemical environment were limited to well-instrumented research sites. Consequently, many of the pandemic studies discussed in Sections 4 and 5 made use of measurements in China, where long-term investment in instrumentation and monitoring to support the development of air quality policy enabled a comprehensive characterization of changing atmospheric composition. This underscores the need to establish more routine, extensive monitoring of reactive gases and aerosols around the world. However, efforts to establish such monitoring are hampered by significant costs, associated with both equipment and personnel to operate state-of-the-art instrumentation in the field. The nascent ACTRIS and ASCENT networks in Europe and the United States, though not established in time to characterize the COVID-19 perturbation, may start to address this need in the future, particularly for aerosols, though more routine radical and VOC measurements will require additional investment. Comprehensive atmospheric chemistry measurements are urgently needed in the Southern Hemisphere (and remote regions worldwide) where emissions and the chemical environment may differ substantially from established sites in North America, Europe, and Asia.

The availability of high-resolution NO_2_ observations from satellites was a key resource to both documenting changes in concentrations across the globe, but more crucially to developing approaches to estimate emissions changes in near real-time. Such estimates, and complementary bottom-up efforts using activity data, are critical in identifying if observed atmospheric responses to changes in emissions are consistent with our understanding of atmospheric chemistry.

The COVID-19 pandemic produced examples of both highly ameliorated and highly degraded local air quality. However, many regions experienced little change in exposure to the central air pollutants of interest: O_3_ and PM_2.5_. Globally, the changes in these constituents and the resulting impact on climate was quite modest. While primary species respond more or less linearly to emissions, the most critical species for global air quality and climate, O_3_ and (secondary) PM_2.5_, respond highly nonlinearly to emissions of a range of precursors. This includes emissions from numerous sectors, including natural sources. Thus, while the COVID-19 pandemic was an unprecedented perturbation to transport emissions in particular, this did not produce a large shift in global atmospheric composition. Accordingly, the experience of the COVID-19 pandemic highlights that clean air policies may be successfully achieved at the local level, but that moderating the impact of short-lived constituents on climate at the global scale requires coherent and multisectoral efforts.
